# Meat Quality in Katerini and Podolian Young Bulls Raised on Pasture: A Comparison between Organic Production Systems in Greek and Italian Environments

**DOI:** 10.3390/ani13193102

**Published:** 2023-10-05

**Authors:** Despoina Karatosidi, Christina Ligda, Maria Antonietta Colonna, Efthymios Avgeris, Simona Tarricone

**Affiliations:** 1 Research Institute of Animal Science, Hellenic Agricultural Organization-DIMITRA, 58100 Giannitsa, Greece; 2 Veterinary Research Institute, Hellenic Agricultural Organization-DIMITRA, 60458 Thessaloniki, Greece; chligda@elgo.gr; 3 Department of Soil, Plant and Food Science, University of Bari Aldo Moro, Via Amendola 165/A, 70125 Bari, Italy; simona.tarricone@uniba.it; 4 Panellinia Enosi Ektropheon Autochthonon Fylon Agrotikon Zoon-PEEAFAZ, S. Sarafi 30, 42100 Trikala, Greece; avgeris.efthimios@gmail.com

**Keywords:** autochthonous breeds, cattle, Katerini, Podolian, meat quality, fatty acid profile, grass-fed bulls

## Abstract

**Simple Summary:**

Katerini cattle is an autochthonous Greek breed considered at high risk of extinction, given the uncontrolled introduction of foreign breeds into local herds and the lack of a national program aiming at monitoring cross-breeding in local populations. As a consequence, Greece has committed to international treaties to create the necessary infrastructure and to prepare a national strategy for the development of actions able to protect indigenous genetic resources and agricultural biodiversity and to participate in cooperation networks, both at a national and global level. This study provides information, which may contribute to the rescue and valorization of the autochthonous Greek Katerini breed through the protection and preservation of biodiversity, as well as an increase in the productivity of farmed animals.

**Abstract:**

Local and typical agri-food products (TAP) are receiving increasing interest from consumers, since they are perceived as genuine, healthy and tasty because they are produced under environmentally friendly farming systems. This has aroused a renewed interest among breeders from the inner regions of Italy and Greece toward autochthonous animal populations, such as Greek Katerini and Italian Podolian cattle. Twenty animals were used, divided into two homogeneous groups of ten subjects per each genotype. Animals were fed only on natural pasture and were slaughtered at 18 months of age. Meat from the Katerini young bulls showed a lower a* value, higher moisture and was leaner, and its fat was richer in n-3 fatty acids and had a better n-6/n-3 ratio. Meat from Podolian young bulls was more tender and showed a higher redness value and a significantly greater MUFA concentration. This preliminary study provides a contribution to the local actors and relevant authorities to develop a conservation program for the endangered Katerini breed based on the nutritional and sensorial characterization of its products.

## 1. Introduction

Local and traditional agri-food products (TAP) are receiving increasing interest from consumers, since they are perceived as genuine, nutritious, healthy and tasty because they are produced under environmentally friendly farming systems. This trend provides new opportunities for the sustainable breeding of local animal populations [1]. 

Extensive livestock farming contributes significantly to the preservation of marginal areas in several ways: it prevents soil erosion and forest fires, improves water infiltration and its retention in the soil and soil fertility, reduces the use of artificial fertilizers and biocides and limits population migration from rural areas to cities [2,3]. Animals are reared with access to grass and pasture during the year; this production system is sustainable and is in line with the preservation of the agro-ecological system. Furthermore, grass-feeding is being frequently used in cow–calf systems, since it contributes to the microbiology, fertility and pedological structure of soil [4]. However, maximization of grazing, as suggested in organic systems, is quite difficult in both Greek and southern Italian environments due to low summer rainfall and poor grassland quality [5].

The composition of the local vegetation is the basis for providing meat with specific nutritional and organoleptic properties, since indigenous grass and pastures are rich in plant secondary compounds, among which some molecules may partially protect valuable polyunsaturated fatty acids from the ruminal biohydrogenation processes [6], and thus, they may be transferred to meat [7]. Therefore, grass-feeding animals play an important role in the transformation of inedible resources into valuable food for human consumption with high nutritional value, thus contributing to the economic development of rural areas [4,8,9]. In this sense, adequate management of pastures and grazing through integrated crop–livestock systems provide many positive effects [10] and improve our knowledge of the relationship between forage resources and animals and their importance to animal productivity and sustainability [11]. In recent decades, Mediterranean grasslands have received little attention from scientific research, as they are perceived as marginal lands, where grazing activity is the only viable solution. However, following correct management, grasslands can become an important feeding source for livestock and can therefore improve their overall productivity [12,13].

In such systems, and particularly in the European Union context, local ruminant breeds represent a valuable genetic resource and are mainly associated with organic and sustainable production systems [14]. Additionally, local breeds, which are well adapted to harsh environments, play a crucial role in the maintenance of traditions, cultural heritage and gastronomy [15,16,17,18]. 

In this context, the interest toward the Katerini cattle, a native breed classified under the risk of extinction, has been regained, and farmers are supported by agro-environmental measures for continuing raising the breed. Beginning in the 2000s, in the rural areas of southern Italy, with a similar ecological environment to Thessaly, farmers focused on the enhancement of Podolian cattle breeding and, in a few years, the number of animals noticeably increased. As both Katerini and Podolian cattle are dual-purpose autochthonous breeds, classified as *Bos taurus primigenius*, they are characterized by comparably low body weight (BW) and muscular appearance (see Supplementary Materials).

While Podolian cattle has been thoroughly studied for its milk and meat production and quality [19,20,21,22,23,24,25] traits, limited information is available for the Greek Katerini cattle breed. Τhe existing literature mainly refers to the historical origin, the phenotypic characteristics and demographic evolution of the breed [26,27]. As the interest in local cattle breeds increases, some information on the genetic characterization of the breed is reported [28]. Furthermore, the analysis of data from the certified abattoirs in Greece, recorded on the ARTEMIS database, the Information System of the Hellenic Agricultural Organization, shows the potential of local cattle breeds, including the Katerini cattle, for the development of the beef sector in Greece [29]. 

Therefore, the present investigation aimed at carrying out a comparative study on the carcass traits and meat quality features of the autochthonous Greek Katerini and Italian Podolian cattle breeds reared under an organic grass-feeding system, typical of the rural areas in which the animals are reared. 

## 2. Materials and Methods

### 2.1. Animal Management and Forage Quality

The trial was carried out on two farms selected in Italy and Greece, respectively, in the Basilicata and Thessaly regions, in relation to the pasture composition. The Italian “Rago” (Latitude 40.281628 N, Longitude 15.896160 E; 650 m asl) and the Greek “Ark Dimou” (Latitude 39.693786 N, Longitude 21.680996 E; 300 m asl) organic farms are located roughly at the same latitude and are focused on meat production.

All animal-related procedures complied with the Directive 2010/63/EU of the European Parliament [30]. A total of 20 calves, 10 Greek Katerini (KAT) and 10 Italian Podolian (POD), born from parents registered in the relevant herdbooks of each breed between November 2020 and January 2021, were included in this study. The calves were reared according to the traditional farming system for the Katerini and Podolian breeds, as they were exclusively milk-fed, suckling from the cows until they reached the age of about 6 months during May. Afterward, the calves were separated from their mothers, weighed and allowed to have free access to pasture. 

The average age of calves for both breeds was 200 + 20 days; the initial weight of the calves was 70 ± 9 and 115 ± 7 kg, respectively, for Katerini and Podolian breeds.

The two groups were raised under an organic extensive grazing system based on local pastures and kept outdoors during the whole year, as, in the selected locations, there were no hazardous environmental conditions, such as snow, high humidity and/or high air temperatures. At the beginning of May 2021, three grass surfaces (15 m^2^ each, 3 × 5 m) were delimited within the grazing areas along an imaginary line corresponding to the diagonal of maximum length, almost equidistant from each other. For each test surface, all the grass was cut during the ante meridiem hours of the day at a height, which simulated that at which the cattle graze, taking care to leave in place the non-palatable species (asphodel, ferula, etc.). The grass collected from the three test areas was promptly weighed. The same grass sampling was carried out in August and October of the same year and in February 2022 to analyze the pasture during each season.

Samples of each plot were ground in a hammer mill with a 1 mm screen and analyzed in triplicate using the AOAC [31] procedures. The fatty acid composition of samples from each ground pasture was determined using the method described below for the meat FA profile. The chemical composition of the pasture in both countries, as well as its fatty acid profile, are mentioned in Table 1.

### 2.2. Slaughter and Sampling Procedures

At the age of 18 months, the animals were slaughtered according to the EU legislation [32]. After fasting for 12 h, with free access to water, young bulls were transported to a local public slaughterhouse and weighed immediately before slaughtering (slaughter weight). The hot carcass, skin and gastrointestinal tract were weighed. Carcasses were hung and chilled at 0–4 °C (80–82% relative humidity) for 24 h and then re-weighed [33]. The refrigerated carcasses were split into two halves across the mid-line; the right side was divided into different cuts (neck, shoulder, leg, steaks, brisket) and weighed separately. The meat cuts were also stored at 4 °C for further 24 h and then dissected into tissue components (lean, dissectible fat and bone), which were weighed. From each carcass, the 9–11th rib section of the *Longissimus lumborum* muscle was removed and transported from the slaughterhouse to the laboratory under refrigerated conditions.

The pH values were measured on the *Longissimus lumborum* (LL) muscle 1 h after the slaughter (pH1) and after 24 h refrigeration (pH24) using a portable instrument (Eutech Instruments XS PH110, Singapore, Singapore) with a Hamilton Double Pored penetrating electrode. 

### 2.3. Physical Parameters of Meat from the Longissimus lumborum Muscle

Meat color (L* = lightness, a* = redness, b* = yellowness) was determined using a Hunter Lab MiniscanTM XE Spectrophotometer (Model 4500/L, 45/0 LAV, 3.20 cm diameter aperture, 10 standard observer, focusing at 25 mm, illuminant D65/10; Hunter Associates Laboratory Inc., Reston, VA, USA). Three readings were taken for each sample by placing the instrument on different meat-handling surfaces. The instrument was normalized to a standard white tile before performing the analysis (Y = 92.8, x = 0.3162 and y = 0.3322). The reflectance measurements were performed after the samples were allowed to oxygenate in the air for at least 30 min to take stable measurements.

Homogeneous samples (approximately 5 cm thick) were cut from the *Longissimus lumborum* muscle and cooked in a ventilated electric oven at 165 °C until an internal temperature of 75 °C was reached in the center of the meat sample, as recorded by a thermocouple [33]. To calculate the percentage of water loss during cooking, meat samples were weighed before and after cooking. Raw and cooked LL muscle samples (25.4 mm in diameter, with fibers perpendicular to the direction of the blade) were assessed for shear force (in triplicate) using a WB device, with a cutting speed of 200 mm/min and shearing until the sample was completely cut. The shear force value reported for each steak was the average value for all the evaluated cores.

The rest of the *Longissimus lumborum* muscle was homogenized in a blender and stored for 1 h at 4 °C until subsequent analysis for chemical composition and intramuscular fatty acid profile. 

### 2.4. Chemical Composition and Fatty Acid Analyses of Meat from the Longissimus lumborum Muscle

To analyze the chemical composition of meat, representative sub-samples of LL muscles were homogenized, and AOAC procedures [31] were used to assess the moisture, ether extract, protein and ash.

Fat was extracted according to the method suggested by Folch et al. [34], using a 2:1 chloroform/methanol (*v*/*v*) solution to determine the fatty acid profile. The fatty acids were then methylated using a KOH/methanol 2N solution [35] and analyzed with a gas chromatograph (Shimadzu GC-17A with FID detector, Kyoto, Japan) equipped with a flame ionization detector and fitted with a PBX-70 capillary column (60 m, 0.25 internal diameter and 0.25 μm film thickness, SGE) and using a split/splitless injection system (split ratio of 1:30) and helium as a carrier gas at a flow rate of 1.5 mL/min. The injection port and detector were maintained at 245 and 280 °C, respectively. The column oven temperature was programmed for 5 min at 135 °C, followed by an increase of 3 °C/min to 210 °C, and finally held at 210 °C for 20 min. Individual fatty acids were identified by comparing their retention times with those of a standard fatty acid mix Matreya. The conjugated linoleic acid (CLA) content in meat was assessed as previously described [36].

The food risk factors of meat were determined by calculating the atherogenic (AI) and thrombogenic (TI) indices [37]:

AI = [(C12:0 + 4 × C14:0 + C16:0)] ÷ [ΣMUFA + Σn-6 + Σn-3]

TI = [(C14:0 + C16:0 + C18:0)] ÷ [(0.5 × ΣMUFA + 0.5 × Σn-6 + 3 × Σn-3 + Σn-3)/Σn-6]

where MUFA are monounsaturated fatty acids.

### 2.5. Statistical Analysis

The data were analyzed for variance (ANOVA) using the GLM procedure of SAS software (9.3) [38]. One-way ANOVA was used to analyze the data, and the main effect tested was breed. Significance was declared at *p* < 0.05; the results are reported as least-squares mean and standard error of the mean (SEM).

## 3. Results

### 3.1. Slaughtering and Carcass Traits of Young Bulls

Table 2 shows the main carcass traits of KAT and POD young bulls. The slaughter weight and the hot and cold carcass weights were significantly (*p* < 0.01) greater in the POD group; the hot and cold dressing percentages were significantly (*p* < 0.01) higher in KAT young bulls. 

The percentages of head and skin were higher (*p* < 0.05) in the POD group, while the gastrointestinal tract (*p* < 0.01) was greater in KAT young bulls.

Finally, the drip loss was markedly higher in the POD group (*p* < 0.05).

The results of the dissection of the right half carcass are shown in Table 3. The weight of the right-side half carcass was significantly higher (*p* < 0.01) in the POD group. Among the commercial cuts, the percentages of the ham (*p* < 0.01) and the shoulder (*p* < 0.05) were greater in POD young bulls, whereas KAT bulls showed higher (*p* < 0.01) percentages of kidney and perirenal fat.

Table 4 shows the dissection data of the *Longissimus lumborum* muscle of young bulls. The relative weight of the loin of the POD group was significantly greater (*p* < 0.01), while no differences were found in the percentage of lean, fat and bone.

### 3.2. Physical Properties of Meat from the Longissimus lumborum Muscle

The results of the physical properties of meat are presented in Table 5. No differences between breeds were found for pH at 24 h postmortem, L* and b* indices. Meat from Podolian young bulls showed greater redness (*p* < 0.01) as compared to the Katerini breed. The WBS of raw KAT meat samples was significantly higher (*p* < 0.05) as compared to POD, while no differences were observed either for the cooking loss or for the WBS of cooked meat. 

### 3.3. Chemical Composition and Fatty Acid Profile of Meat from the Longissimus lumborum Muscle

The chemical composition of the meat is shown in Table 6. Meat from the POD group had a significantly lower (*p* < 0.05) moisture and N-free extract content and a higher (*p* < 0.01) percentage of fat.

Data referring to the fatty acid profile of intramuscular fat in POD and KAT meat samples are presented in Table 7. Several differences in individual fatty acids between the two breeds were found: meat from Katerini young bulls was characterized by a significantly higher percentage (*p* < 0.01) of C15:0, C15:1, C17:1, C18:3n3 and eicosapentaenoic acid (EPA). Furthermore, higher concentrations (*p* < 0.05) of C18:0, C14:1, conjugated linoleic acid (CLA) (9Z,11E), C22:5n3 were also found.

On the contrary, meat from Podolian young bulls showed a higher (*p* < 0.01) concentration of C16:1n7 and C18:1n9c.

Therefore, KAT meat had a lower (*p* < 0.01) value of total MUFA and UFA, which determined a lower value of the saturated/monounsaturated ratio (*p* < 0.05). 

Total n-3 was higher (*p* < 0.05) in KAT meat; as a consequence, the n-6/n-3 ratio was significantly lower (*p* < 0.01).

## 4. Discussion

Livestock production experienced a radical change in the 1960s in all the European countries; there has been a progressive evolution from traditional farming systems, based on small herds of local breeds grazing on natural vegetation, to the introduction of specialized breeds farmed in confined systems in order to increase productivity and farm income. This has led to an important concern over modern animal breeding: the risk of genetic erosion and loss of animal biodiversity [39]. In fact, many local breeds are suffering uncontrolled cross-breeding, which has led to a severe reduction in the number of animals [40]. 

In southern Italian marginal areas, sustainable animal-friendly methods, including organic production, are performed using autochthonous breeds in loose housing conditions (i.e., outdoor access, free-range) and feeding with natural resources, since local breeds show better utilization of pasture than highly specialized genotypes and provide milk and meat, which are very appreciated both by locals and visitors, becoming a destination for food tourism. 

Since the 1990s, under the increasing awareness of the importance of livestock diversity and the global strategy for the management of farm animal genetic resources of FAO [41], many efforts have been targeted toward the conservation and sustainable utilization of local breeds. Specific interest has been given to certain local cattle breeds, which were severely threatened by extinction [42]. The conservation of genetic resources of cattle breeds is important for several reasons, such as the preservation of cultural and historical values of a country, the maintenance of genetic diversity and the adaptation to changing environmental conditions. 

Although our trial was performed in lands with similar pasture availability, productivity and floristic composition, in order to minimize the differences between the two areas, we are aware that some uncontrollable factors may have occurred during the study, such as exceptional and sporadic weather events; therefore, further investigations are necessary to replicate these preliminary findings, which are a starting point for future research.

So far, few literature works are available on the productive performances and meat quality traits of Katerini cattle [15], while several studies have been carried out on Podolian cattle farmed in southern Italian regions, such as Basilicata [19,20,43] and Apulia [5,21,24], under different farming systems. In our study, the slaughtering weight was generally lower as compared to the findings reported by other authors for young bulls slaughtered at the same age, which received feed supplementation in addition to grazing during the finishing period, while the dressing percentage was quite similar [5,21]. 

With regard to the dissection of the half carcass, Podolian young bulls showed a higher percentage of the shoulder and pelvic limb, even though this result was lower as compared to our previous findings [43]. It may be hypothesized that the genetic selection programs carried out in the last decade on the Podolian breed may have led to a better conformation of the carcass with a higher percentage of first grade cuts.

No differences between breeds were observed for meat pH, L* and b* values, and WBS. Meat from Podolian young bulls showed higher redness as compared to the Katerini breed. Several factors influence the consumer perception of meat quality, which are strongly influenced by intrinsic (age and carcass weight at slaughter, carcass fatness, sex, genotype) and extrinsic aspects, mainly the animal feeding and production system [44]. Grass-feeding is known to affect the color of meat and fat as compared to concentrate-fed animals, since concentrates have lower β-carotene and other pigment concentrations, which affect the color of the whole muscular tissue. Furthermore, grass-fed animals have more muscle myoglobin due to the greater exercise performed by animals as compared to feedlot counterparts [45], which leads to higher proportions of oxidative fibers [46]. In this study, the meat color features from Podolian young bulls were similar to those reported in previous experiments on this breed [25,47]. Meat color, tenderness, flavor, juiciness and shelf-life are influenced by pH [48]. No differences between breeds were observed for the pH values of meat; therefore, variances in meat color may be attributable to genetic differences between Podolian and Katerini cattle, as well as to the pasture characteristics and environmental conditions.

Based on these preliminary results, meat from Podolian young bulls was more tender as compared to the Katerini breed, which was probably related to the higher intramuscular fat content of meat from Podolian young bulls. However, the cooking process leveled the differences between breeds in terms of the meat shear force. Tenderness is a multifactorial quality criterion, characterized by great variability, and it is therefore difficult to control or predict. The appreciation of beef tenderness is generally positively correlated with the intramuscular content of fat; in fact, lean meats are often perceived as having reduced tenderness, flavor and juiciness [49]. Moreover, the cooking process influences the overall eating quality of meat, depending on the temperature and duration of cooking, which is hardly comparable among the different studies [50,51,52].

The fatty acid profile of intramuscular fat of beef is of great interest due to its implication for the risk of cardiovascular disease [53] and its positive relationship with the sensory properties of beef meat, such as the aroma, flavor, juiciness and tenderness [54]. The amount of beef intramuscular fat and its composition are impacted by the cattle breed or genetic variation, fat deposition and feeding on grass rather than concentrates [55].

Many differences between breeds were found for the individual fatty acids; focusing on the fatty acids important for human health, meat from the Katerini breed showed a higher concentration of α-linolenic (C18:3n-3), eicosapentaenoic (EPA), conjugated linoleic acid (CLA; 9Z, 11E) and docosapentaenoic C22:5n-3 acid, and consequently, a two-fold greater amount of n-3 fatty acids and a better n-6/n-3 ratio. De La Torre et al. 2006 [56] stated that the CLA content in beef is related to the rate of fattening: animals with lean meat showed higher CLA concentration as compared to those with greater levels of intramuscular fat. This process may have also occurred in our trial, since meat from Katerini young bulls, which was leaner, showed a higher CLA content. These results seem to be very promising in terms of the potential benefits for human health [57]; however, further investigation is needed to confirm these preliminary results, since the literature is lacking in information on the Katerini cattle breed.

In this study, Podolian meat showed a greater MUFA concentration, while no differences between breeds were detected for the SFA and PUFA contents. The results observed in this study for the fatty acid profile of Podolian meat are quite similar to those reported in the literature [19,24,43]. There is general agreement on the fact that the genotype is the major source of variation in meat fatty acid composition [58]. Cuvelier et al. [59] reported that higher intramuscular fat content in Limousin and Aberdeen Angus bulls was associated with a higher SFA and MUFA content, whereas the PUFA concentration varied only to a small extent. This fact seems to be mainly due to the preferential incorporation of PUFA into the phospholipids within the cell membranes, whereas SFA and MUFA are deposited into the triacylglycerol fraction, which increases with the intramuscular fat content. The higher PUFA content in some breeds may be explained by the more oxidative nature of the muscle, with consequently more cell membranes and more phospholipids, as indicated by the large differences between breeds in terms of enzyme activity related to the metabolic type of muscular fiber [58].

PUFA/SFA is an index normally used to assess the impact of diet on cardiovascular health (CVH). It hypothesizes that all PUFAs in the diet can depress low-density lipoprotein cholesterol (LDL-C) and lower the levels of serum cholesterol, whereas all SFAs contribute to high levels of serum cholesterol. Thus, the higher this ratio, the more positive the effect [60]. For beef, the typical value of the PUFA/SFA ratio is quite low (around 0.1), except for grass-fed animals, where this ratio is much higher; as a matter of fact, in this study, the PUFA/SFA ratio ranged from 0.31 to 0.35, respectively, for Podolian and Katerini young bull meat. Furthermore, the n-6/n-3 ratio for grass-fed beef was also beneficially low (around 3), reflecting the significant amount of desirable n-3 PUFA in green pasture. This increased content of PUFA may be explained by the presence of secondary plant metabolites during ruminal digestion, which inhibits microbial biohydrogenation [61].

## 5. Conclusions

Katerini meat showed valuable characteristics, since it was lean and with an optimal nutritional profile of intramuscular fat. Our findings contribute to the efforts toward the valorization of the breed through utilization of its desirable properties. Further research is necessary to understand the genetic background of these characteristics and develop appropriate breeding and management strategies. Future investigations are needed to study the influence of finishing systems on growth performances and meat quality characteristics of these local cattle populations. Local and collective efforts, including stakeholders, should be encouraged, aiming at the certification of the specific quality of meat from the Katerini breed.

## Figures and Tables

**Table 1:** Chemical and fatty acid composition of the pasture (mean ± SE).

	Pasture							
	Italy				Greece			
Variable	May	August	October	February	May	August	October	February
Floristic composition (%)								
Grass	81.90 ± 5.83	62.63 ± 5.83	81.83 ± 5.83	74.24 ± 5.83	81.68 ± 5.83	61.26 ± 5.83	81.95 ± 5.83	78.47 ± 5.83
Legumes	5.92 ± 2.27	4.36 ± 2.27	7.99 ± 2.27	6.89 ± 2.27	6.36 ± 2.27	4.71 ± 2.27	7.81 ± 2.27	6.64 ± 2.27
Composites	7.24 ± 5.52	23.15 ± 5.52	5.24 ± 5.52	6.27 ± 5.52	6.40 ± 5.52	24.28 ± 5.52	5.88 ± 5.52	4.89 ± 5.52
Others	4.94 ± 3.02	9.86 ± 3.02	4.94 ± 3.02	12.60 ± 3.02	5.71 ± 3.02	9.75 ± 3.02	4.36 ± 3.02	10.00 ± 3.02
Chemical composition (%)								
Dry matter	14.00 ± 6.43	66.72 ± 6.43	29.98 ± 6.43	54.52 ± 6.43	17.27 ± 6.43	61.82 ± 6.43	26.97 ± 6.43	27.45 ± 6.43
Protein	6.79 ± 1.71	5.18 ± 1.71	9.06 ± 1.71	4.12 ± 1.71	6.39 ± 1.71	10.96 ± 1.71	13.40 ± 1.71	3.98 ± 1.71
Fat	2.52 ± 0.23	1.20 ± 0.23	1.34 ± 0.23	1.58 ± 0.23	1.27 ± 0.23	1.58 ± 0.23	1.35 ± 0.23	1.66 ± 0.23
Ash	13.30 ± 2.67	9.98 ± 2.67	13.29 ± 2.67	6.52 ± 2.67	7.28 ± 2.67	8.06 ± 2.67	16.78 ± 2.67	7.15 ± 2.67
Crude fiber	32.62 ± 4.12	33.95 ± 4.12	31.98 ± 4.12	31.78 ± 4.12	44.50 ± 4.12	30.58 ± 4.12	21.04 ± 4.12	38.90 ± 4.12
N-free extract	44.77 ± 4.78	49.69 ± 4.78	44.33 ± 4.78	56.00 ± 4.78	40.56 ± 4.78	48.82 ± 4.78	47.43 ± 4.78	48.31 ± 4.78
NDF ^1^	36.97 ± 3.89	59.74 ± 3.89	38.68 ± 3.89	68.32 ± 3.89	46.93 ± 3.89	56.10 ± 3.89	45.35 ± 3.89	73.57 ± 3.89
ADF ^1^	12.85 ± 5.91	10.14 ± 5.91	22.31 ± 5.91	40.93 ± 5.91	10.89 ± 5.91	13.07 ± 5.91	15.56 ± 5.91	29.86 ± 5.91
ADL ^1^	44.76 ± 0.89	1.43 ± 0.89	13.68 ± 0.89	12.50 ± 0.89	40.55 ± 0.89	2.65 ± 0.89	1.05 ± 0.89	8.65 ± 0.89
Fatty acid composition (% FA methyl esters)								
C14:0 (myristic)	0.65 ± 0.09	0.29 ± 0.09	0.47 ± 0.09	0.11 ± 0.09	0.57 ± 0.09	0.31 ± 0.09	0.37 ± 0.09	0.17 ± 0.09
C16:0 (palmitic)	13.29 ± 1.37	14.08 ± 1.37	11.80 ± 1.37	14.47 ± 1.37	12.95 ± 1.37	13.74 ± 1.37	14.46 ± 1.37	13.81 ± 1.37
C18:0 (stearic)	1.26 ± 0.25	1.91 ± 0.25	2.09 ± 0.25	2.28 ± 0.25	1.92 ± 0.25	2.34 ± 0.25	2.02 ± 0.25	2.67 ± 0.25
C18:1 n-9, *cis* 9 (oleic)	15.49 ± 2.44	12.55 ± 2.44	26.25 ± 2.44	16.41 ± 2.44	14.56 ± 2.44	12.47 ± 2.44	24.22 ± 2.44	17.04 ± 2.44
C18:2 n-6 (linoleic)	13.09 ± 2.98	17.87 ± 2.98	19.84 ± 2.98	14.73 ± 2.98	13.05 ± 2.98	17.61 ± 2.98	18.98 ± 2.98	15.01 ± 2.98
C18:3 n-3 (α-linolenic)	2.54 ± 0.51	2.10 ± 0.51	2.58 ± 0.51	2.61 ± 0.51	2.24 ± 0.51	2.22 ± 0.51	2.74 ± 0.51	2.63 ± 0.51
C22:0 (behenic)	1.45 ± 0.38	2.88 ± 0.38	1.74 ± 0.38	1.85 ± 0.38	0.89 ± 0.38	1.14 ± 0.38	1.98 ± 0.38	2.21 ± 0.38
C22: 5 n-3 (DPA)	0.13 ± 0.02	0.15 ± 0.02	0.17 ± 0.02	0.12 ± 0.02	0.16 ± 0.02	0.19 ± 0.02	0.12 ± 0.02	0.17 ± 0.02
C22:6 n-3 (DHA)	0.12 ± 0.02	0.14 ± 0.02	0.14 ± 0.02	0.17 ± 0.02	0.18 ± 0.02	0.15 ± 0.02	0.18 ± 0.02	0.17 ± 0.02

^1^ NDF, neutral detergent fiber; ADF, acid detergent fiber; ADL, acid detergent lignin.

**Table 2:** Slaughtering data of Katerini and Podolian young bulls.

	KAT	POD	SEM ^1^ (DF = 18)	*p*-Value
Slaughter weight (kg)	216.00 ^B^	334.85 ^A^	18.611	0.009
Hot carcass weight (kg)	116.60 ^B^	164.20 ^A^	12.823	0.009
Cold carcass weight (kg)	115.10 ^B^	161.00 ^A^	12.322	0.008
Hot right half carcass (kg)	58.51 ^B^	82.57 ^A^	7.314	0.007
Hot left half carcass (kg)	58.09 ^B^	81.40 ^A^	7.141	0.007
Cold right half carcass (kg)	57.56 ^B^	80.85 ^A^	7.185	0.008
Cold left half carcass (kg)	57.43 ^B^	80.07 ^A^	6.976	0.006
Hot dressing percentage (%)	53.89 ^A^	48.91 ^B^	3.439	0.047
Cold dressing percentage (%)	53.15 ^A^	47.99 ^B^	3.491	0.008
Drip loss (%)	1.41 ^b^	1.87 ^a^	0.361	0.029
Head ^2^	4.09 ^b^	4.76 ^a^	0.381	0.027
Skin ^2^	6.49 ^b^	8.23 ^a^	0.959	0.025
Gastrointestinal tract ^2^	5.10 ^A^	3.54 ^B^	0.307	0.009
Shins ^2^	2.34	2.66	0.299	0.063

^1^ SEM: standard error of means, DF: degrees of freedom; ^2^ % on body weight; ^a, b^ *p* < 0.05; ^A, B^ *p* < 0.01.

**Table 3:** Dissection data of the right half carcass of young bulls (%).

	KAT	POD	SEM ^1^ (DF = 18)	*p*-Value
Right-side weight (kg)	55.34 ^B^	81.15 ^A^	6.542	0.008
Ham	25.96 ^B^	30.87 ^A^	1.919	0.006
Shoulder	15.06 ^b^	16.46 ^a^	0.786	0.032
Loin + filet	11.76	10.64	1.508	0.071
Brisket	14.78	13.99	1.624	0.082
Neck with bone	17.20	15.26	1.849	0.074
Belly	12.01	10.67	1.759	0.086
Tail	1.40	1.34	0.249	0.091
Kidney	0.96 ^A^	0.44 ^B^	0.098	0.005
Perirenal fat	0.85 ^A^	0.34 ^B^	0.119	0.004

^1^ SEM: standard error of means, DF: degrees of freedom; ^a, b^ *p* < 0.05; ^A, B^ *p* < 0.01.

**Table 4:** Anatomical dissection (%) of the *Longissimus lumborum* muscle of young bulls.

	KAT	POD	SEM ^1^ (DF = 18)	*p*-Value
*Longissimus lumborum* muscle (kg)	4.15 ^B^	7.03 ^A^	0.694	0.006
Lean (%)	58.44	63.14	11.451	0.063
Dissectible fat (%)	11.59	15.99	5.759	0.072
Bone (%)	19.08	20.89	4.635	0.078

^1^ SEM: standard error of means, DF: degrees of freedom; ^A, B^ *p* < 0.01.

**Table 5:** Physical parameters of meat from the *Longissimus lumborum* muscle of young bulls.

	KAT	POD	SEM ^1^ (DF = 18)	*p*-Value
pH 24	5.56	5.51	0.215	0.084
L* (lightness)	39.35	36.52	1.770	0.094
a* (redness)	12.88 ^B^	16.61 ^A^	0.942	0.008
b* (yellowness)	11.54	12.61	0.649	0.063
WBS raw meat (kg/cm^2^)	2.88 ^a^	2.17 ^b^	0.049	0.035
WBS cooked meat (kg/cm^2^)	5.55	5.36	2.373	0.062
Cooking loss (%)	24.40	22.06	7.848	0.082

^1^ SEM: standard error of means, DF: degrees of freedom; ^a, b^ *p* < 0.05; ^A, B^ *p* < 0.01.

**Table 6:** Meat chemical composition (%).

	KAT	POD	SEM ^1^ (DF = 18)	*p*-Value
Moisture	75.36 ^a^	73.69 ^b^	1.060	0.035
Protein	20.42	21.47	0.805	1.02
Fat	1.71 ^B^	3.11 ^A^	0.411	0.005
Ash	1.25	1.16	0.094	0.981
N-free extract	1.24 ^A^	0.57 ^B^	0.241	0.008

^1^ SEM: standard error of means, DF: degrees of freedom; ^a, b^ *p* < 0.05; ^A, B^ *p* < 0.01.

**Table 7:** Fatty acid composition (% total FA methyl esters) of meat from the *Longissimus lumborum* muscle.

Fatty Acids	KAT	POD	SEM ^1^ (DF = 18)	*p*-Value
C14:0 (myristic)	2.89	1.86	0.880	0.152
C15:0 (pentadecanoic)	0.84 ^A^	0.32 ^B^	0.170	0.006
C16:0 (palmitic)	20.61	20.17	2.070	0.856
C17:0 (heptadecanoic)	0.60	0.55	0.080	0.074
C18:0 (stearic)	20.57 ^a^	15.71 ^b^	2.330	0.02
C20:0 (eicosanoic)	0.59	0.49	0.080	0.078
C14:1 (tetradecenoic)	0.20 ^a^	0.12 ^b^	0.040	0.037
C15:1(pentadecanoic)	0.31 ^A^	0.17 ^B^	0.040	0.008
C16:1 n-7 (palmitoleic)	1.38 ^B^	2.18 ^A^	0.280	0.003
C17:1 (cis-10-Heptadecenoic acid)	1.04 ^A^	0.74 ^B^	0.090	0.008
C18:1 n-7 (cis-vaccenic)	2.31	3.17	1.410	0.074
C18:1 n-9t (elaidic)	0.38	0.45	0.080	0.083
C18:1 n-9c (oleic)	22.32 ^B^	34.13 ^A^	2.440	0.007
C18:2 n-6t	0.34	0.14	0.240	0.076
C18:2 n-6c (linoleic)	7.50	8.47	2.930	0.069
CLA_(9Z,11E)_ (conjugated linoleic acid)	0.21 ^a^	0.09 ^b^	0.050	0.039
CLA_(10E,12Z)_ (conjugated linoleic acid)	0.08	0.18	0.070	0.082
C18:3 n-3 (α-linolenic)	2.23 ^A^	0.35 ^B^	0.470	0.007
C18:3 n-6 (γ-linolenic)	0.16	0.02	0.120	0.095
C20:2 n-6 (eicosadienoic)	0.07	0.06	0.060	0.086
C20:3 n-3 (eicosatrienoic)	3.73	1.78	1.450	0.073
C20:3 n-6 (dihomo-γ-linolenic)	0.69	0.43	0.320	0.081
C20:4 n-3 (eicosatetraenoic)	0.12	0.11	0.070	0.079
C20:4 n-6 (ARA)	0.02	0.00	0.030	0.091
C20:5 n-3 (EPA)	0.50 ^A^	0.09 ^B^	0.040	0.009
C22:5 n-3 (DPA)	1.23 ^a^	0.34 ^b^	0.440	0.039
C22:6 n-3 (DHA)	0.19	0.20	0.080	0.083
Other acids	9.36	7.68	2.945	0.082
Saturated fatty acids (SFAs)	46.24	39.09	5.035	0.076
Monounsaturated fatty acids (MUFAs)	28.32 ^B^	40.96 ^A^	3.241	0.008
Polyunsaturated fatty acids (PUFAs)	16.16	12.26	5.569	0.069
Unsaturated fatty acids (UFAs)	44.49 ^b^	53.23 ^a^	4.930	0.031
Total n-3	6.92 ^a^	2.68 ^b^	2.232	0.038
Total n-6	9.25	9.59	3.316	0.074
n-6/n-3	1.35 ^B^	3.66 ^A^	0.286	0.008
PUFA/SFA	0.35	0.31	0.090	1.001
SFA/PUFA	3.24	3.40	1.133	0.099
UFA/SFA	0.99 ^b^	1.36 ^a^	0.208	0.039
Atherogenic index	0.75	0.52	0.194	0.098
Thrombogenic index	1.97	1.40	0.390	0.087

^1^ SEM: standard error of means, DF: degrees of freedom; SFAs: saturated fatty acids (sum of C10:0 + C14:0 + C15:0 + C16:0 + C17:0 + C18:0 + C20:0); MUFAs: monounsaturated fatty acids (sum of C14:1 + C15:1 + C16:1 c9 + C17:1 c10+ C18:1 t11 + C18:1 t9 + C18:1 t10 + C18:1 c9); Total n-6 (sum of C18:2 c9;c12 + C18:2 c9;t11 + C18:3 + C20:3 + C20:4 + C22:6); Total n-3 (sum of C18:3 + C20:3 + C20:4 + C20:5 + C22:5 + C22:6); PUFAs: polyunsaturated fatty acids (sum of n-6 +n-3); UFAs: unsaturated fatty acids (sum of MUFAs + PUFAs); ^a, b^ *p* < 0.05; ^A, B^ *p* < 0.01.

## Data Availability

The data presented in this study are available upon reasonable request from the corresponding author.

## Supplementary Materials

The following supporting information can be downloaded at: https://www.mdpi.com/article/10.3390/ani13193102/s1, Figure S1: Geographical origin of Katerini and Podolian autochthonous cattle breeds. Generated by the authors. (a). Podolian, (b) Katerini; Table S1: Main characteristics of the Katerini and Podolian breeds. References [62, 63, 64 and 65] are cited in the Supplementary Materials.
