# Supplementary material for: Meat Quality in Katerini and Podolian Young Bulls Raised on Pasture: A Comparison between Organic Production Systems in Greek and Italian Environments
Source: Animals (Basel). 2023 Oct 5;13(19):3102. doi: 10.3390/ani13193102 (PMC10571818; doi:10.3390/ani13193102)
Supplement: Supplementary file 1 [file animals-13-03102-s001.zip › animals-2628426-supplementary.pdf]

**ANNEX 1**

Katerini and Podolian populations derive from the *Bos primigenius* or *Uro*, the first documented bovine; its domestication seems to have occurred four centuries Before Christ in the Middle East. There are two theories about the origin of these two breeds [1]. According to the former, they derived from cattle that came to South Europe in 452 B.C. following Huns who, along their way from Mongolia, passed through the Ukrainian steppe, which is considered as their birthplace. However, according to another theory, in the isle of Crete (Greece) there was long-horn cattle which can be identified as *Bos primigenius*.

Recent assessments indicate that there are 36.652 heads of Podolian cattle in South Italy, registered in the Herdbook [2] and only 825 of the Katerini breed [3].

The Podolian cattle are spread throughout an area that mainly covers the inland territories of Southern peninsular Italy (Abruzzo, Basilicata, Calabria, Molise and Apulia) [2,4]. The Podolian breed has been used in the past, mainly for its labor capacity and secondarily for meat and milk production. Its milk is ideal for producing the famous "caciocavallo" cheese [5].

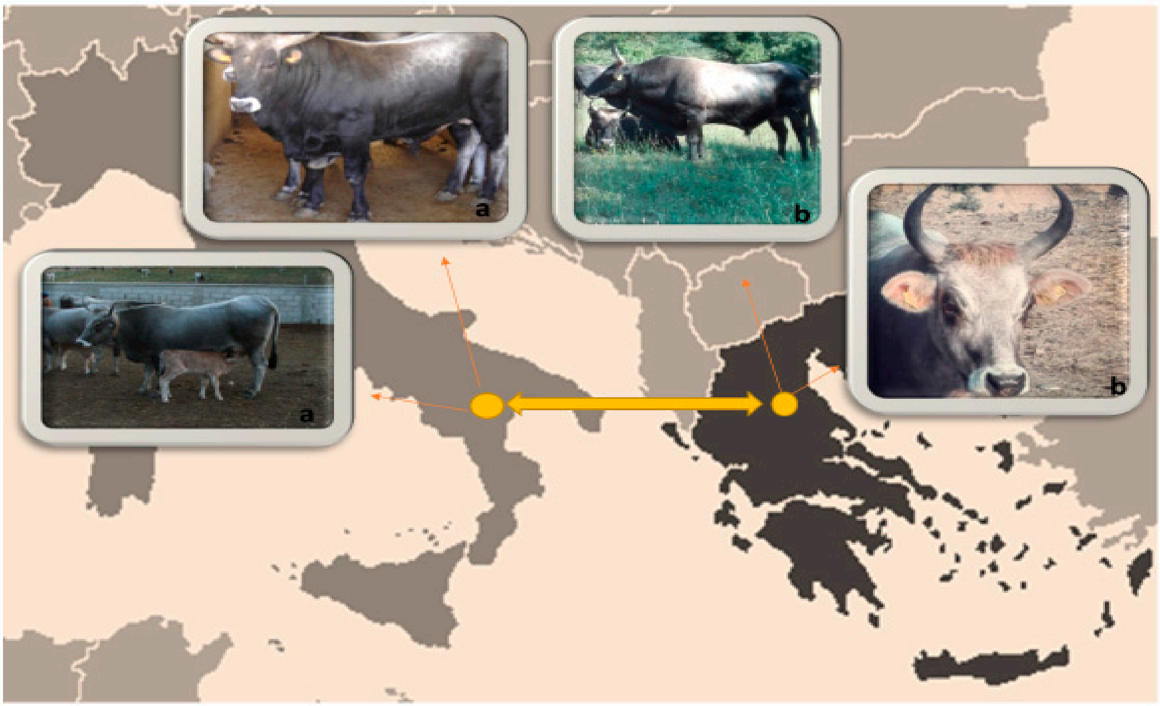


**Figure 1.** Geographical origin of Katerini and Podolian autochthonous cattle breeds. Generated by the authors. (a). Podolian, (b) Katerini.

The Katerini cattle breed is small sized with an average wither height of 125 cm and 113 cm for the males and females, respectively. The head is large with long lyra shaped horns. The neck is medium to short and strong. The basic colour is grey, but it ranges from black to brown. The average weight (at 24 months of age) is 375 kg for the males and 280 kg for the females. The breed is characterized by a white ring around the nose. In general, the body conformation is of a strong draft animal of the steppe type. The few remaining animals are raised in the region of Thessaly (central Greece) [6], where there is an increase of female/herd (females registered in the Herdbook) in the last 10 years from 328/4 to 825/8 [3].

However, due to the rise and spread of agricultural mechanization, the selection of these two breeds was oriented mainly towards meat production and, to a lesser extent, towards dairy production, particularly in certain areas. As far as the reproductive aspect is concerned, age at first calving is rather advanced (about 3 years). This is primarily due to the breed's harsh habitat, especially due to extreme heat of the summer months, with the amount of available food resources being decreased; thus, the growth rate of younger animals is slower. Nevertheless, these types of cattle develop a long reproductive life, breeding for over ten years with an average time span of fifteen months between calvings. For the most part, calvings are spontaneous and they are concentrated during late winter. Calves suckle for at least six months [2]. The

calves are slaughtered in order to be sold for meat at around 15-18 months with weights ranging around 300-350 kg (in Greece, the slaughtering weight is lower and around 250 kg).

**Table 1.** Main characteristics of the Katerini and Podolian breeds.

| | KAT | POD |
|---|---|---|
| Date of herdbook establishment | 1999 | 1988 |
| Productive attitude | meat | meat/milk |
| **Morphology information at 24 months** (average) | | |
| Wither height (cm) | 123 ♂/113 ♀ | 159 ♂/144 ♀ |
| Weight (kg) | 375 ♂/280 ♀ | 500 ♂/350 ♀ |
| **Performance information** (average) | | |
| Parturition interval (days) | 450 ± 10 | 400 ± 10 |
| Age at first parturition (month) | 34 ± 0.5 | 36 ± 1 |
| Birth weight (kg) | 25 ± 3 | 40 ± 5 |
| Age of maturity (month) | 24 ♂/20 ♀ | 24 ♂/20 ♀ |
| Length of productive life (years) | 9 | 12 |

## References


1. Senczuk, G., Mastrangelo, S., Ajmone-Marsan, P. et al. On the origin and diversification of Podolian cattle breeds: testing scenarios of European colonization using genome-wide SNP data. *Genet Sel Evol* **2021**, *53*, 48. https://doi.org/10.1186/s12711-021-00639-w
2. ANABIC. Standard della Razza Podolica. **2007**. Available online http://www.anabic.it/servizio_tecnico/podolica.pdf (accessed on 10 August 2023).
3. DAD-IS. Available online: http://www.fao.org/dad-is/en/ (accessed on 10 August 2023).
4. Marsico, G.; Rasulo, A.; Forcelli, M.G.; Tarricone, S.; Pinto, F.; Cagnetta, P.; Basile, G. Aspetti quanti-qualitativi delle carni di vitelloni podolici puri ed F1 alimentati con razioni contenenti buccette d'uva. *Taurus Sp*. **2008**, 6, pp. 15-31 (in Italian).
5. Tarricone, S.; Marsico, G.; Ragni, M.l Forcelli, M.G.; Pinto, F.; Vicenti, A.; Rasulo, A. Chemical and fatty acid characteristics of meat of Podolian bulls slaughtered at different ages. *Ital. J. Anim. Sci*. **2009**, 8, suppl. 2: 575.
6. Ligda, C. Status of Podolic cattle in Greece. In Proceedings of *Sulle tracce delle Podoliche* **2009**, Conference, *Taurus* **2009**, 101-110.
